# SPIDer: *Saccharomyces *protein-protein interaction database

**DOI:** 10.1186/1471-2105-7-S5-S16

**Published:** 2006-12-18

**Authors:** Xiaomei Wu, Lei Zhu, Jie Guo, Cong Fu, Hongjun Zhou, Dong Dong, Zhenbo Li, Da-Yong Zhang, Kui Lin

**Affiliations:** 1 MOE Key Laboratory for Biodiversity Science and Ecological Engineering and College of Life Sciences, Beijing Normal University, Beijing 100875, China

## Abstract

**Background:**

Since proteins perform their functions by interacting with one another and with other biomolecules, reconstructing a map of the protein-protein interactions of a cell, experimentally or computationally, is an important first step toward understanding cellular function and machinery of a proteome. Solely derived from the Gene Ontology (GO), we have defined an effective method of reconstructing a yeast protein interaction network by measuring relative specificity similarity (RSS) between two GO terms.

**Description:**

Based on the RSS method, here, we introduce a predicted *Saccharomyces *protein-protein interaction database called SPIDer. It houses a gold standard positive dataset (GSP) with high confidence level that covered 79.2% of the high-quality interaction dataset. Our predicted protein-protein interaction network reconstructed from the GSPs consists of 92 257 interactions among 3600 proteins, and forms 23 connected components. It also provides general links to connect predicted protein-protein interactions with three other databases, DIP, BIND and MIPS. An Internet-based interface provides users with fast and convenient access to protein-protein interactions based on various search features (searching by protein information, GO term information or sequence similarity). In addition, the RSS value of two GO terms in the same ontology, and the inter-member interactions in a list of proteins of interest or in a protein complex could be retrieved. Furthermore, the database presents a user-friendly graphical interface which is created dynamically for visualizing an interaction sub-network. The database is accessible at .

**Conclusion:**

SPIDer is a public database server for protein-protein interactions based on the yeast genome. It provides a variety of search options and graphical visualization of an interaction network. In particular, it will be very useful for the study of inter-member interactions among a list of proteins, especially the protein complex. In addition, based on the predicted interaction dataset, researchers could analyze the whole interaction network and associate the network topology with gene/protein properties based on a global or local topology view.

## Background

Protein-protein interactions play a key role in many biological processes and therefore, are increasingly forming the focus of studies in understanding protein function and cellular behaviour [1,2]. Genome-scale protein interaction networks have now been determined experimentally for several model organisms [3-12]. However, these interaction datasets are often incomplete and noisy [13,14]. Therefore, several *in silico *interaction predictions have been designed as complementary to existing experimental approaches, which are derived from gene context analysis, such as gene neighbourhood, gene fusion events, phylogenetic profiles and correlated mRNA expression patterns. Detailed reviews of these methods can be found elsewhere [15,16]. Currently, the interaction predictions have been improved by integrating different genomic features based on a single probabilistic framework in yeast [17,18] and human [19].

**Figure 1:**
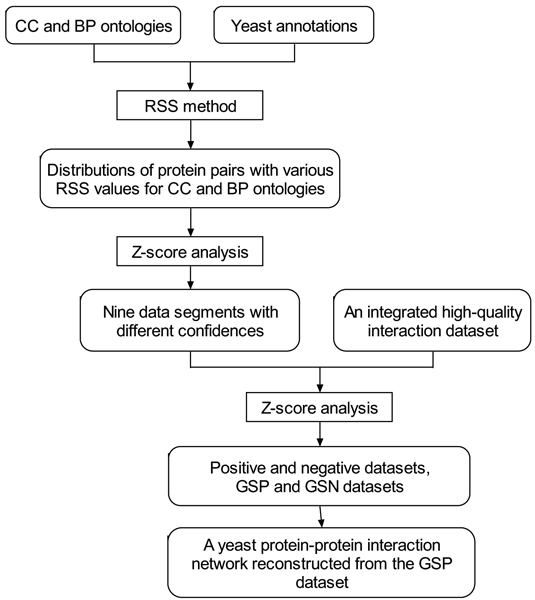
**Schematic representation of the GO-based prediction of protein-protein interaction network**. It includes four steps: (1) Using both CC and BP ontologies and their respective annotations, the distributions of protein pairs with various RSS values for the two ontologies were obtained. (2) Then a Z-score analysis was applied to draw the statistical significance of the quality scoring system and the nine data segments with different combinations of confidences were obtained. (3) To evaluate the RSS method, an integrated high-quality interaction dataset was applied. Based on a Z-score analysis, a positive dataset and a negative dataset were selected. Moreover, a gold standard positive (GSP) dataset with the highest level of confidence and a gold standard negative (GSN) dataset with the lowest level of confidence were derived. (4) Finally, a yeast protein-protein interaction network was reconstructed from the GSP dataset. The RSS method, a new metric for semantic similarity to score the degree of the functional association between two different proteins by comparing the relative specificity of pairs of GO terms assigned to them in similarity within a GO DAG [21].

Originating from the Gene Ontology (GO), which organizes biological information for molecular function (MF), biological process (BP) and cellular component (CC) for different model organisms [20], we have defined a new metric of relative specificity similarity (RSS) to predict the functional association of two proteins [21]. Using both CC and BP ontologies and their respective annotations from Organelle DB [22], we derived a gold standard positive (GSP) dataset with the highest confidence of RSS values and then reconstructed a protein-protein interaction network [21]. A schematic explanation of the prediction of interaction network is shown in Figure 1.

Using our computational method, we have now developed a web-accessible database, *Saccharomyces *Protein-protein Interaction Database (SPIDer). This database contains the predicted yeast protein-protein interaction dataset, which is based on a new release of GO and the annotations derived from SGD [23]. Moreover, it provides users with a graphical interface to visualize an interaction sub-network for a list of proteins of interest.

### Construction and content

We used the MySQL database system and Perl scripting language to develop and implement our database. The graphical view of network was designed using the Graphviz tool [24]. Our server runs on the Linux operating system. The content of the database is as follows.

#### GSP dataset

SPIDer houses the data for yeast protein-protein interactions (GSP dataset) solely derived from the GO and yeast annotations by measuring RSS between two GO terms at the genome level (Figure 1 and Ref [21]). In previous work, we used the September 2004 release of GO and yeast annotations from Organelle DB [22] to obtain a GSP dataset, which covers 78% of the integrated high-quality interaction dataset (valid experimental interactions) [21]. Here, we used the March 2006 release of the GO and yeast protein annotations downloaded from SGD submitted on March 31, 2006. The derived GSP dataset is proved to have a high confidence level, as it covers 79.2% of the high-quality interaction dataset which was used in the previous work [21]. The current GSP dataset consists of 92 257 interactions encompassing 3600 proteins, and the reconstructed interaction network forms 23 connected components, out of which the largest contains 3527 proteins and 92 084 interactions.

A detailed help page explaining the contents of the database is available online at the website.

#### Cross references to other databases

SPIDer provides general links to connect predicted protein-protein interactions with four other related datasets derived from three databases, namely, DIP [25], BIND [26] and MIPS [27]. Both DIP (the release of April 2, 2006 was used here) and BIND (the release of May 21, 2005) are important databases that contain information on protein-protein interactions. MIPS provides all annotated and genome-scale protein interactions (the release of January 18, 2005), and also compiles data on protein complexes (the release of June 20, 2005), which are converted to binary interactions using the matrix model. As a result, there are 15 296 interactions in GSPs that have at least one connection to the four datasets, out of which 2951, 1431, 1135 and 14 452 interactions in our database have cross references to DIP, BIND, MIPS binary interactions and MIPS complexes, respectively.

### Utility

Datasets in SPIDer can be accessed through a variety of search features.

**Figure 2:**
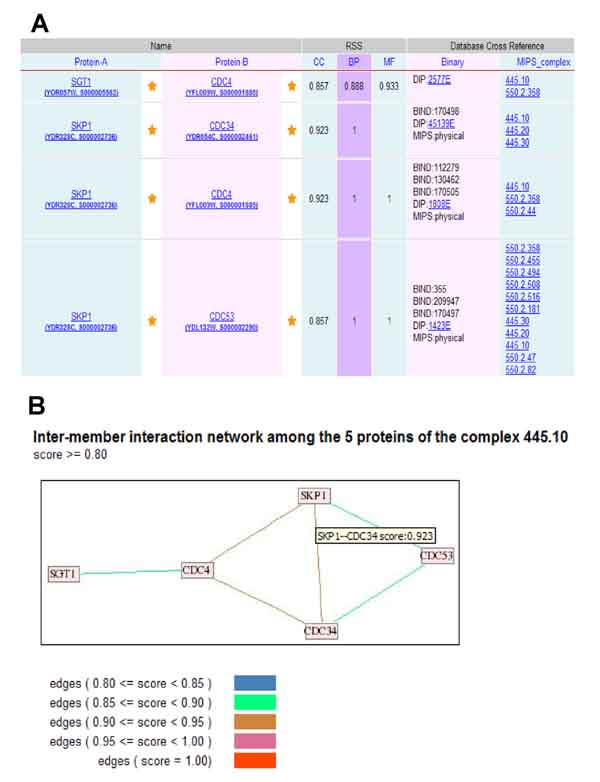
**Sample query output and graph representation from SPIDer**. (A) Partial results of inter-member interactions from the query for the SCF-CDC4 complex (search by inputting the MIPS identifier '445.10'). The table of cross-member interactions has the following information: the information for two interacting proteins, their RSS values on the three ontologies (CC, BP and MF), the cross references to other binary interaction datasets (DIP, BIND and MIPS physical interactions) and the MIPS complexes, as well as the hyperlinks to the evidence information. This table is designed to be clickable and provides hyperlinks of the proteins to SGD. Both DIP binary interactions and MIPS complexes have links to their home pages. (B) A graphical representation of the inter-member interaction network of the SCF-CDC4 complex using the default score cutoff. Nodes and edges represent proteins and cross-member interactions, respectively. Each node is labeled standard gene name. Each edge is depicted in colour representing the RSS score, which is the minimum of the BP and CC RSS values. In addition, the ORF name and primary SGD identifier are obtained when the cursor is positioned over a node, and score value over an edge (an example of the interaction between CDC34 and SKP1 is shown). Five different scores of edges in the legend are depicted in five colours.

(i) Searching by protein information: given an SGD [28] identifier, a group of interactors of the query protein is returned. The interaction table is designed to be clickable and thus directs users to more detailed information, such as cross references to other databases, and hyperlinks to the pages with evidence information retrieved from the datasets we integrated. Before visualizing the network of queried interactions dynamically, two filtering criteria – the RSS score cutoff with default 0.85 and interaction step with default 1 – can be set to navigate the final graphical display of the network. No result to any given query may be due to the absence of the query protein from the GSP dataset. This search option also allows users to specify a keyword of standard gene name, open reading frame (ORF) name or protein description and to retrieve a list of SGD entries that match the keyword. Then, one or multiple proteins of interest can be selected and used in turn as query protein(s) for retrieving their interactors. Moreover, the selection of multiple query proteins provides access to the extraction and visualization of their inter-member interaction relationship.

(ii) Searching by GO term information: by entering a GO term identifier or a keyword of the term description, users can retrieve a list of proteins annotated on the term(s).

(iii) Retrieving the RSS value of two GO terms in the same ontology by entering their identifiers. This option allows users to access the maximum RSS value computed between the two GO terms.

(iv) Browsing inter-member interactions by entering a MIPS complex identifier or a list of proteins: we provide users with a search facility to extract the inter-member interactions of a list of proteins of interest. It allows inputting a MIPS complex identifier, and also a list of gene names, ORF names or SGD identifiers either by simply pasting them into the text box, or by uploading a plain file where proteins are separated by a space or a newline. For example, in order to identify the topology of SCF-CDC4 complex (MIPS identifier 445.10; containing five members), the identifier is entered. Figure 2 shows the search results and a graphical view of the sub-network of the complex.

(v) Searching by sequence similarity: this search feature facilitates users to retrieve protein sequences matching a query sequence or its fragment. The results are returned as a list of proteins ordered by the BLAST e-value.

## Discussion

The yeast protein-protein interaction dataset will continue to be updated using the new release of GO and yeast annotations in SGD. Furthermore, in the future, we intend that our database will provide more detailed analysis results of the whole interaction network, which are useful in analyzing and understanding yeast proteome. We also expect to reconstruct the maps of interactions from other completely sequenced genomes with high-quality GO-based annotations for functional genomic research.

## Conclusion

SPIDer is an open and public database server for protein-protein interactions which are solely derived from the Gene Ontology (GO), based on the yeast genome. It provides users with convenient access to protein interactions based on various search features. In particular, it allows users to analyze the potential inter-member interactions among a list of proteins of interest, which is especially useful for the analysis of protein complexes. Furthermore, it presents a graphical interface for visualizing an interaction sub-network, facilitating users to associate the network topology with gene/protein properties based on a global or local topology view.

## Availability and requirements

The database is freely accessed on the internet [29]. The complete gold standard positive dataset is on request by contacting with the corresponding author.

## List of abbreviations used

SGD: *Saccharomyces *Genome Database

DIP: Database of Interacting Proteins

BIND: Biomolecular Interaction Network Database

MIPS: Munich Information Center for Protein Sequences

## Authors' contributions

XMW was responsible for the development and construction of the database and drafted the manuscript, while LZ designed and implemented the web interface. JG provided essential suggestions for designing the database and helped in drafting the manuscript. KL designed and coordinated the work. CF, HJZ, DD, ZBL and DYZ made their great efforts in acquiring and analyzing data, and in testing the database and the web interface. All authors read and approved the final manuscript.
